# The mechanisms behind heatstroke-induced intestinal damage

**DOI:** 10.1038/s41420-024-02210-0

**Published:** 2024-10-28

**Authors:** Minshu Sun, Qin Li, Zhimin Zou, Jian Liu, Zhengtao Gu, Li Li

**Affiliations:** 1 Department of Treatment Center For Traumatic Injuries, The Third Affiliated Hospital of Southern Medical University, Guangzhou, Guangdong China; 2 Academy of Orthopedics·Guangdong Province, Orthopedic Hospital of Guangdong Province, Guangdong Provincial Key Laboratory of Bone and Joint Degenerative Diseases, The Third Affiliated Hospital, Southern Medical University, Guangzhou, Guangdong China; 3 Department of Intensive Care Unit, The Sixth Affiliated Hospital, Sun Yat-sen University, Guangzhou, Guangdong China

**Keywords:** Acute inflammation, Cell death, Mechanisms of disease

## Abstract

With the frequent occurrence of heatwaves, heatstroke (HS) is expected to become one of the main causes of global death. Being a multi-organized disease, HS can result in circulatory disturbance and systemic inflammatory response, with the gastrointestinal tract being one of the primary organs affected. Intestinal damage plays an initiating and promoting role in HS. Multiple pathways result in damage to the integrity of the intestinal epithelial barrier due to heat stress and hypoxia brought on by blood distribution. This usually leads to intestinal leakage as well as the infiltration and metastasis of toxins and pathogenic bacteria in the intestinal cavity, which will eventually cause inflammation in the whole body. A large number of studies have shown that intestinal damage after HS involves the body’s stress response, disruption of oxidative balance, disorder of tight junction proteins, massive cell death, and microbial imbalance. Based on these damage mechanisms, protecting the intestinal barrier and regulating the body’s inflammatory and immune responses are effective treatment strategies. To better understand the pathophysiology of this complex process, this review aims to outline the potential processes and possible therapeutic strategies for intestinal damage after HS in recent years.

## Facts

- The intestines are one of the first organs to be affected after heatstroke.
- Intestinal damage is an important cause of death from heatstroke.
- Damage to the intestinal barrier involves multiple mechanisms.

## Open questions

- What are the mechanisms that lead to intestinal damage after heatstroke?
- What are the key molecules and mechanisms that cause intestinal cell necroptosis in the context of heatstroke pathogenesis?
- What changes will occur in intestinal microorganisms after heatstroke? What impact will these changes have on the intestines?
- How to prevent intestinal damage after heatstroke?

## Introduction

Heatstroke (HS) is a life-threatening disease, and its incidence and mortality are rising annually as the effects of global warming become more severe. HS can be divided into two types based on its triggering factors, which are classic heatstroke and exertional heatstroke [1, 2]. According to the statistics, the mortality rates of exertional heatstroke and classic heatstroke can reach 26.5% and 63.2%, respectively, under intensive care [3]. One important reason is that, currently, there is no effective clinical treatment for HS [4]. Thus, it is necessary to carry out research on mechanisms. The current view is that the mechanism of heat injury is due to direct damage from heat and systemic inflammatory response syndrome (SIRS) caused by heat stress and endotoxin leakage. This pathological process also refers to HS as a “like-sepsis reaction” [5, 6].

HS can cause damage to multiple organs and tissues, among which the intestine is one of the most important [7]. It is believed that the disruption of the intestinal barrier contributes to and initiates HS. Under HS, massive intestinal epithelium loss occurs, which increases intestinal permeability and bacterial translocation. At the same time, the endotoxins in the intestinal flora penetrate into the circulation and induce SIRS [5, 8, 9]. Therefore, the gastrointestinal tract has long been considered the “motor” of multiple organ failure and a key factor in various critical illnesses [10, 11]. Severe intestinal epithelial damage is also considered to be the main cause of death from HS [12]. Through a large number of studies, it has been found that the mechanism of intestinal damage is complex and interrelated. The body’s stress response, oxidative damage, destruction of intestinal tight junction (TJ) proteins, excessive cell death, and imbalance of intestinal flora are all challenges faced by the intestine under HS. Therefore, this review aims to summarize the research on the mechanisms related to intestinal damage caused by HS in recent years. Moreover, some intervention strategies based on mechanisms from recent years will also be involved.

## Manifestations of intestinal damage in HS

When the core body temperature rises above 42 °C, both baboons and mice can exhibit a large loss of intestinal epithelium, and severe damage occurs in both the small intestine and the large intestine, manifested as villous stroma broadening, focal necrosis, edema, and congestion. In addition, it is accompanied by enhanced apoptotic cell death in the lamina propria and inflammatory cell infiltration [13–16].

After intestinal damage, one of the most obvious manifestations is increased intestinal permeability. In a healthy state, the paracellular transport involved in TJ mainly includes the pore pathway and the leakage pathway. However, pathological findings in experimental models confirm that material transport cannot be finely controlled under heat stress, and harmful substances such as endotoxins will cross the barrier and be absorbed into the blood, causing serious damage to the body. Therefore, endotoxin has been shown to be a key factor in HS fatalities [17, 18].

In addition, in fatal cases of HS, there is extensive bleeding and necrosis of the intestine [19]. According to a rat study, HS can induce disseminated intravascular coagulation [20]. In HS, inflammation and cell death caused by heat stress can activate the coagulation cascade through the extrinsic and intrinsic pathways, ultimately leading to the formation of an “inflammatory thrombus” [21]. However, despite the complex interplay between coagulation and inflammation, activation of coagulation is not a prerequisite for cell injury or organ damage [22]. Experiments have shown that the expression and release of tissue factor were increased in the jejunal tissue of the HS model [13]. This tissue factor initiates the extrinsic coagulation pathway, leading to thrombin generation and fibrin formation, and is confirmed as the main initiator of coagulation activation in HS [19, 23]. However, inhibition of tissue factor does not modulate systemic inflammatory responses [22]. Therefore, coagulopathy is more likely to be a mechanism other than inflammation that causes intestinal damage.

## Mechanisms of intestinal damage

### Heat stress response

It is vital to describe the body’s reaction to relieve stress during HS to comprehend the mechanism causing intestinal damage. Heat stress response (HSR) is a core component of the body’s defense against dangerous environmental heat. It not only plays a role in HS but also has a protective effect on dealing with other stress situations, such as pathology and physiological stimulation. The inducing factors of HSR are related to the misfolding of proteins. Normally, proteins maintain their natural structure and functions at appropriate temperatures. However, under HS conditions, rising temperatures can cause proteins to misfold and form aggregates, which serve as intracellular signals to induce HSR [24]. The onset of HSR function requires the participation of special proteins, heat shock proteins (HSPs). Under HS conditions, the expression of these proteins is induced by heat shock transcription factor (HSF) and increases rapidly, especially HSF1 and HSF2 [25]. Activated HSPs can assist in protein refolding, thereby reducing the load of incorrect proteins [26]. At present, HSP70 is the most widely studied, and it is also the most conserved molecular chaperone protein. When HSF1 is activated, the transcription and synthesis of HSP70 will be initiated, which will then block the nuclear factor-κB (NF-κB) pathway of immune cells and reduce the release of pro-inflammatory cytokines [27]. There is evidence showing that the expression of HSP70 protects cells from damage associated with heat stress [28], including stabilizing junctional proteins and repairing the cytoskeleton [29], assisting in the upregulation of occludin [30], and suppressing intestinal cell apoptosis [31]. Consequently, circumstances that impair HSR may make people more vulnerable to HS.

In addition, in recent years, it has been proposed that HSP response may be necessary for the body to acquire heat acclimation (HA) [32, 33]. Numerous studies have shown that HA leads to a significant upregulation of HSP70 and HSP72 in humans and animals [34–36]. Increased HSPs can reduce inflammatory responses to improve tolerance to heat stress and prevent intestinal epithelial damage [32]. At the same time, in human epithelial cells (Caco-2), it was found that upregulation of HSP70 increased occludin levels and reduced permeability in vivo [37]. This effect may increase resistance to gut-associated endotoxin translocation, further mitigating intestinal damage [38]. Besides, HA can also reshape the dominant intestinal microbiota to enhance the integrity of the intestinal barrier [39]. Therefore, HA is considered the best strategy to prevent HS [40].

In addition to the intracellular responses mentioned above, stress can also trigger the release of HSPs into the extracellular environment. Unlike the anti-inflammatory effect inside the cell, it seems to play a more immune-enhancing role outside the cell [41, 42]. Under extreme heat stress, HSPs can be released from necrotic cells [43] and through the exosome pathway [44]. These released proteins can promote inflammation by interacting with specific cell surface receptors and leading to the transcription and release of cytokines such as interleukin-1β (IL-1β), tumor necrosis factor-α (TNF-α), and IL-6 [45–47]. In short, HSP is not only involved in anti-inflammatory and cell death prevention in the HS process. The released HSP is also the danger signal of the innate immune system.

### Endoplasmic reticulum stress

After HS, a large number of misfolded proteins accumulate in the body, triggering endoplasmic reticulum stress (ERS) in cells. [48, 49] It has been demonstrated that unfolded protein response (UPR), a signal transduction pathway network to reduce the ERS, consists of three endoplasmic reticulum-related pathways, which are PKR-like ER-resident kinase, activated transcription factor 6, and inositol-requiring enzyme 1/XboX binding protein 1 [43]. These three pathways are considered to be closely related to the inflammatory response and apoptosis. In the HS model, both protein misfolding and excessive production of reactive oxidative species (ROS) can stimulate this stress response [50, 51]. The activated UPR can limit protein synthesis and increase HSP70, which inhibits the NF-κB pathway and reduces inflammation [27]. However, excessive activation of the UPR will have adverse effects on the body. The activation of NF-κB and NOD-like receptor pyrin domain-containing protein-3 leads to intestinal inflammatory response [52, 53]. Furthermore, the apoptosis triggered by UPR is associated with HS-induced intestinal cell death. Some reports prove that CCAAT/enhancer-binding protein homologous protein, a key marker of ERS-mediated apoptosis, participates in this process. Although apoptosis can play a protective role under HS and help restore cell function, excessive apoptosis can disrupt intestinal homeostasis.

In addition, the secretory capacity of intestinal epithelial cells (IECs) depends on endoplasmic reticulum homeostasis. One of the important reasons is the high sensitivity of goblet cells to ERS [54, 55]. Goblet cells have the ability to generate mucus, which forms an uninterrupted mucus barrier that shields the cells from luminal microbes inside the intestine [56]. The normal functioning of this mucosal barrier is inseparable from the mucin-2 [57]. In HS conditions, local intestinal inflammation caused by ERS brings about goblet cell dysfunction, and mucin-2 secretion is reduced [15, 58]. This situation will cause mucus barrier damage and lead to disease progression [54]. To sum up, in the intestinal injury caused by HS, the role of ERS mainly involves three aspects: apoptosis, inflammation, and the impact on goblet cells. Notably, in HS, excessive production of ROS can be transmitted to the endoplasmic reticulum through receptors located on the mitochondrial membrane, triggering ERS. Therefore, some researchers are committed to finding the intersection between oxidative stress and ERS [48]. Recent studies have also revealed the involvement of ROS in the initiation of NF-κB signaling [59]. It seems that ROS not only acts as an activator of ERS, but also activates UPR-related inflammatory pathways. Therefore, ROS/ERS/NF-κB is an important reason for the aggravation of intestinal injury under HS.

### The oxidative stress response

Cell viability is largely dependent on the equilibrium between oxidation and antioxidants. However, when the core temperature of a patient suffering from HS rises above 40 °C, this balance will be destroyed. A large number of studies have shown that HS will cause high levels of ROS [9, 60, 61]. The generated ROS can cause damage to tissues through various pathways. There are currently two main mechanisms for ROS production. First of all, high temperatures can stimulate the production of ROS by affecting the integrity of mitochondrial membranes and their electron transport chains [62]. Furthermore, the body redistributes blood flow in order to better dissipate heat, leading to local ischemia and hypoxia in the internal organs, thereby promoting the production of ROS. Thus, localized hypoxia and ischemia represent yet another major source of ROS generation [63]. Excess ROS can impair intestinal barrier function by reducing epithelial cell viability and damaging intestinal TJs [64]. Alternatively, the production of ROS is also inextricably linked to cell apoptosis. Studies have confirmed that oxidative stress related to heat stress can cause apoptosis in pig small intestine cells [65]. In recent years, new discoveries have been made about the signaling pathways involved in oxidative stress in HS. The work of Jin Yu et al. [60] proposed that the increase in oxidative stress is related to the activation of the mitogen-activated protein kinase (MAPK) signaling pathway. The activation of Jun N-terminal kinase and p38 may lead to intestinal damage caused by HS. Subsequently, Yanan Liu and Gao Yi et al. further confirmed this hypothesis and proposed the role of the lysosome-mitochondria apoptosis pathway in it [9, 61]. After HS stimulates a large accumulation of ROS, they will then mediate the activation of p38 and Jun N-terminal kinase, cause lysosomal damage, and finally induce cell apoptosis by activating caspase-3(Ref. [60]). In contrast, programmed cell death and intestinal damage caused by HS can be effectively reversed when ROS production is reduced, or antioxidants such as misoprostol are used, but the mechanisms involved are currently unknown [66, 67]. Therefore, the oxidative stress response may be an important link in heat stress injury and act upstream of apoptosis.

### Regulation of tight junction

The physical barrier formed by TJ is crucial to intestinal health, which is composed of claudins, occludins, and the intracellular plaque proteins zonula occludens (ZO-1, ZO-2, and ZO-3) [68]. As a signaling center, it is regulated by a variety of intracellular signals, including different isoenzyme forms of protein kinase C [69], protein kinase A [70], MAPK [71], etc. Studies have shown that upon heat stress, multiple kinases and phosphatases will phosphorylate the TJ proteins, resulting in reduced interaction of occludins and claudins with zona-occludins, thereby affecting intestinal permeability [72, 73]. Some experts believe that the phosphorylation of TJ proteins may be the result of endotoxin leakage and the activation of pro-inflammatory cytokines during heat stress [74]. This process involves the outer cytoskeletal ring surrounding the epithelial cells, which is composed of actin and myosin II, also known as the perijunctional actomyosin ring. This skeleton ring connects claudins and occludin via ZO proteins. When the myosin light chain is phosphorylated by myosin light chain kinase (MLCK), this ring will be contracted, thus generating tension on the TJ proteins, affecting the sealing effect between cells and leading to an increase in intestinal permeability [75–77]., Besides, a study on modulation of the expression of ZO-1 found that MLCK has a close relationship with it, which inhibits the stable expression of ZO-1 [72]. Later, a previous study performed by Du et al. [78]. reported that inhibition of MLCK can reduce HS-induced intestinal permeability, intestinal cell apoptosis, and protect the intestinal mucosal barrier. This experiment further proves that MLCK is an important molecule for regulating TJ permeability. Similar to this mechanism, protein kinase C can also increase intestinal permeability by phosphorylating the myosin light chain [18].

**Fig. 1:**
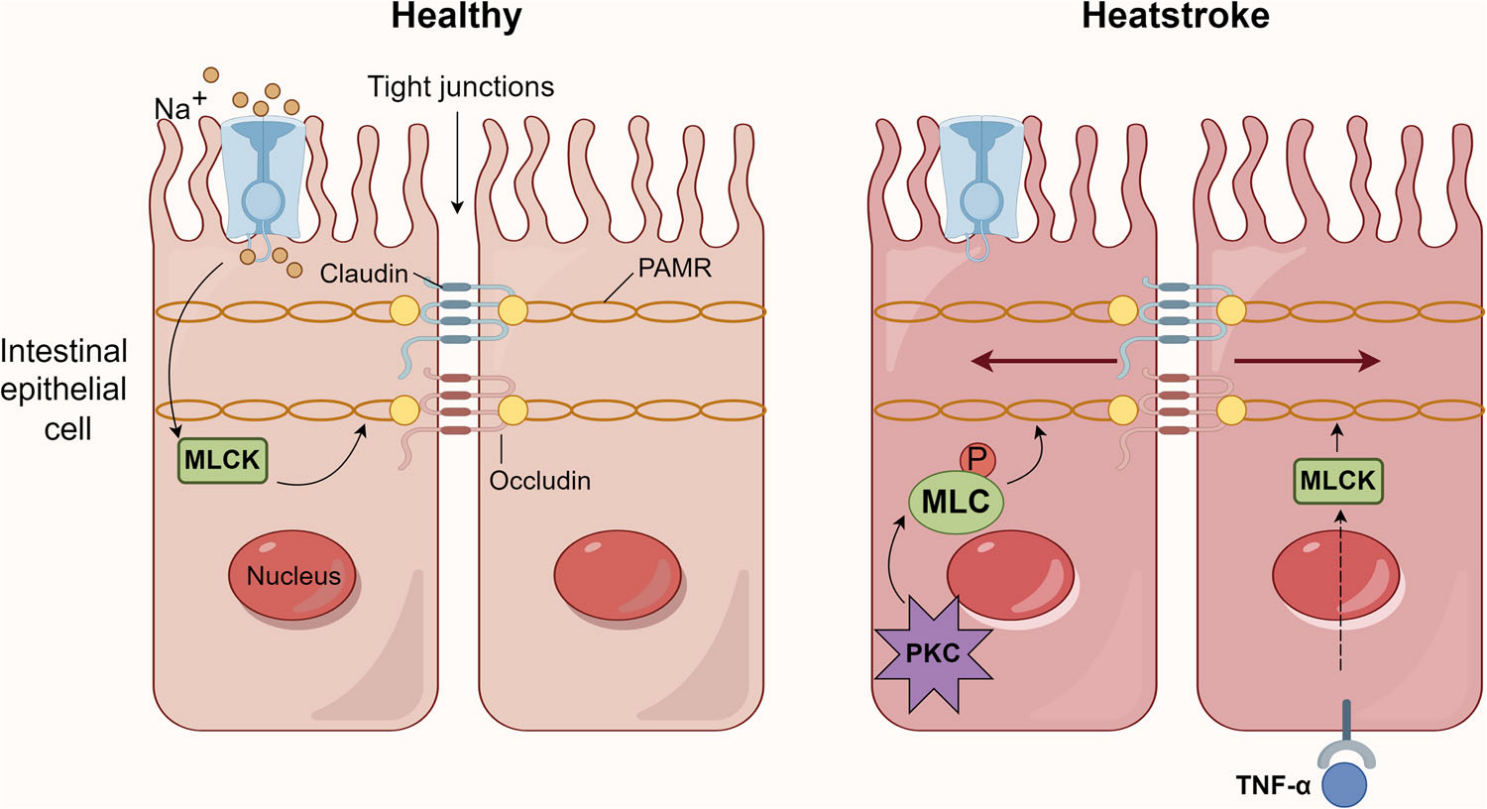
The structure and regulation of TJ under physiological conditions. PAMR is related to the sealing of TJ, which is connected to the tight junction proteins Claudin and Occludin and forms the intestinal epithelial barrier. Studies have found that MLCK and MLC are involved in regulating PAMR and thereby affecting barrier permeability. Under normal physiological conditions, Na^+^/glucose cotransport activates MLCK and phosphorylates MLC, which can cause the contraction of the skeleton ring and generate tension on the TJ, thus loosening the seal between cells and increasing the permeability of the barrier. Under HS conditions, on the one hand, the PKC-MLC pathway will act, leading to the contraction of PAMR. On the other hand, under HS conditions, a large amount of HSP70 is produced, which binds to TLR4 and promotes the secretion of TNF-α. Increased pro-inflammatory factors upregulate the expression of MLCK by activating the NF-κB signaling pathway.

It is well known that the activation of MLCK is related to various factors, for example, inflammatory pathways. After HS, the HSR takes effect, resulting in increased expression of HSP. During this process, one of the important molecules, HSP70, can bind to Toll-like receptors 4 (TLR4), a type of receptor located on the cell membrane surface, to trigger an inflammatory response and stimulate the body to secrete and synthesize pro-inflammatory cytokines such as TNF-α and IL-6, ultimately activating the NF-κB pathway and further amplifying the inflammatory response [79]. Notably, this NF-κB pathway can ultimately damage the intestinal epithelial barrier by upregulating the expression of MLCK [80–82] (Fig.1). It is also established that the MAPK signaling pathway is associated with this enzyme. As an upstream molecule of MLCK, ERK can promote cytoskeletal rearrangement by activating the MLCK and Myosin IIB signaling pathways [83, 84]. Taken together, MLCK displays diversity in signaling pathways related to TJ regulation.

With in-depth research on autophagy, the correlation between programmed cell death and TJ has also been discovered in recent years. Studies have found that in HS models, heat stress can mediate the phosphorylation of ERK1/2 through receptor-interacting protein 1 (RIP1; also known as RIPK1) and receptor-interacting protein-3 (RIP3; also known as RIPK3), the key signaling molecules in necroptosis, thereby regulating the expression of TJ proteins [71]. In summary, a growing body of evidence demonstrates that the signaling pathways and regulatory processes involved in TJ protein expression are extremely complex, but it remains unclear whether HS directly or indirectly causes TJ dysfunction.

### Macrophage interaction with the intestinal barrier

It has been suggested in recent years that macrophages interact with IECs in addition to serving as immune cells that defend the intestine [85, 86]. Most resident intestinal macrophages continuously recruit monocytes from the blood to renew and maintain their macrophage pool [87]. Among the recruited cells, most monocytes differentiate into M2 macrophages (alternatively activated), which exhibit an anti-inflammatory phenotype. Conversely, if recruited during inflammation, monocytes will exhibit a pro-inflammatory phenotype and produce pro-inflammatory cytokines, commonly referred to as M1 macrophages (classically activated) [88–90]. Studies have indicated that distinct macrophage phenotypes have widely differing effects on the intestinal barrier. The activating M2 macrophages can induce TJ expression and promote the intestinal barrier, while M1 macrophages show the opposite effect [91].When the intestinal barrier is damaged and intestinal permeability increases after HS, endotoxin (i.e., lipopolysaccharides) leaks into the internal environment, and the body is stimulated to produce an immune response. In this situation, not only recruited macrophages polarize to the M1 type, but tissue-resident macrophages also exhibit this characteristic [92]. The actin ring will then be compressed by the M1 macrophages, increasing the barrier’s permeability [91]. Although normalization of macrophage function is not necessary to restore epithelial barrier function, changes in macrophages may aggravate intestinal damage to a certain extent under HS conditions.

Based on this, affecting the polarization direction of macrophages seems to be a therapeutic strategy for HS intestinal damage. In macrophages, different transcription factors regulate the gene expression of macrophages. In the nucleus of M1 macrophages, key transcription factors such as NF-κB, signal transducer and activator of transcription 1 (STAT1), STAT5, and interferon regulatory factor 3 (IRF3) regulate the gene expression of M1 macrophages, while M2 macrophages are regulated by transcription factors such as STAT6, IRF4, peroxisome proliferators-activated receptors δ (PPARδ) and PPARγ [93]. Targeting these transcription factors is one of the methods to regulate the polarization direction of macrophages [94]. Immunosuppressants such as corticosteroids [95, 96] and small-molecule inhibitors of Janus kinase (JAK) [97] can play a therapeutic role in inflammatory bowel disease (IBD) due to this mechanism. Recently, it was found that mesenchymal stem cells (MSCs) can change the phenotype of macrophages during sepsis through secreted transforming growth factor beta (TGF-β), thereby reducing inflammation. TGF-β acts on the Akt/Fox01 pathway to change macrophage phenotype and reduce the level of pro-inflammatory factor [98, 99]. Unfortunately, there is still a large gap in the research on this part of HS. However, it is undeniable that macrophages play an important role in the inflammatory response experienced by the intestine after HS [12], so promoting the polarization of M2 macrophages may be a new therapeutic strategy to control intestinal inflammation.

### Programmed cell death

Excessive activation of programmed cell death (PCD) is a significant reason for intestinal epithelium damage in HS-related intestinal damage, in addition to direct thermal stimulation-induced cell damage. Many studies have confirmed the importance of PCD in maintaining intestinal homeostasis [100–102]. However, a prolonged high temperature can disrupt this balance in HS patients.

#### Necroptosis

**Fig. 2:**
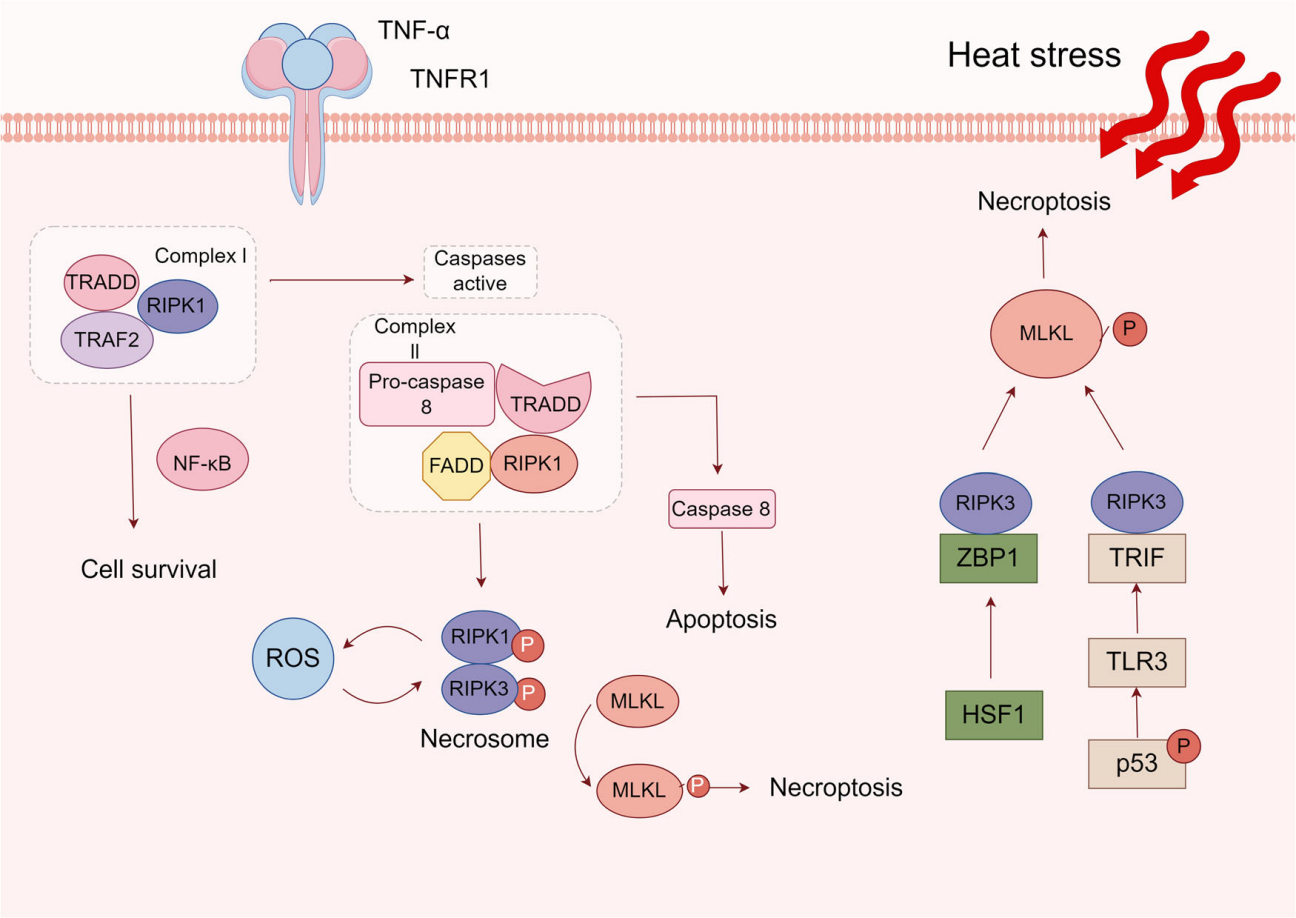
Signal transduction pathways of necroptosis under HS. Necroptosis is often triggered by extracellular stimuli. Its activation relies on a variety of receptors, such as CD95, TNF, and so on. When a ligand (such as TNF-α) binds to a receptor, Complex I can be formed. This complex can promote cell survival through the NF-κB pathway. However, when the interacting components of TNFR1 change, complex I will become complex II. Normally, caspase-8 in Complex II can trigger apoptosis by activating the classic caspase cascade. Conversely, when the signal of caspase-8 is blocked, RIP3 in the cell will be activated to form the necrosome, and then the MLKL will be phosphorylated. Activated MLKL serves as an initiation signal, leading to necroptosis. In recent years, it was found that in heatstroke, the production of ROS and the occurrence of necroptosis promote each other. In addition, there are two RIP1-independent necroptosis pathways, which are mediated by ZBP1 and TLR3, respectively.

Necroptosis, as a newly discovered form of PCD, exhibits unique morphological characteristics that are different from apoptosis or necrosis [103, 104]. When necroptosis occurs, dying cells release chemical signals that trigger an active immune-inflammatory response. Previous studies have shown that high-mobility group box 1 released from necrotic IECs causes liver damage during HS by activating the inflammasome [105]. Therefore, targeting necroptosis is one of the treatment strategies. The activation of necroptosis relies on a variety of receptors, such as TNF [106] and TLR [107]. After HS, a significant increase in the pro-inflammatory cytokine TNF-α can be seen in the plasma [16]. Once the receptor binds to the ligand, the body will have different responses through the formation of the complex [108, 109]. Classical necroptosis requires the participation of RIP1 and RIP3, which then phosphorylate the mixed lineage kinase domain-like protein (MLKL), leading to the occurrence of necroptosis [110]. HS treatment increased the expression levels of RIP1, RIP3, and phosphorylated MLKL both in vitro and in vivo, thus promoting the formation of necrosomes and leading to increased intestinal cell death after HS [66]. Notably, there is an interaction between necroptosis and oxidative stress. After using the ROS scavenger N-acetyl-L-cysteine, it effectively inhibited necroptosis caused by HS, confirming that RIP1/RIP3-mediated cell death depends on the production of ROS [66]. Currently, the relationship between ROS and necroptosis is still unclear. Exploring the crosstalk between the two can provide theoretical and strategic support for alleviating intestinal damage. (Fig. 2).

In addition to the classic necroptosis mode, there are several RIP1-independent necroptosis signaling pathways under HS. Yuan et al. [111]. found that, under heat stress, HSF1 can promote the expression of Z-DNA binding protein 1 and then mediate RIP3-dependent necroptosis. Subsequent experiments also proved that the TLR3/TIR domain-containing adapter-inducing interferon-β/RIP3 pathway, which is dependent on p53, phosphorylates MLKL and initiates necroptosis [16]. In the above two experiments, after inhibiting RIP3, the phosphorylation of MLKL decreased, and cell necrosis was alleviated. Therefore, regardless of the upstream trigger, RIP3 and MLKL are key regulators of necroptosis in HS (Fig. 2).

#### Ferroptosis

In a high-heat environment, disruption of antioxidant balance, accumulation of ROS, shrinkage of mitochondria, and an increase in membrane density are all key features of cell ferroptosis [112]. Under physiological conditions, the System X_c_^-^ is an important system for resisting oxidative stress. It is involved in the synthesis of the reducing agent glutathione (GSH) and glutathione peroxidases (GPXs) in the body [113]. For this reason, System X_c_^-^/GSH/GPX4 is an important pathway for inhibiting ferroptosis [114–116].

**Fig. 3:**
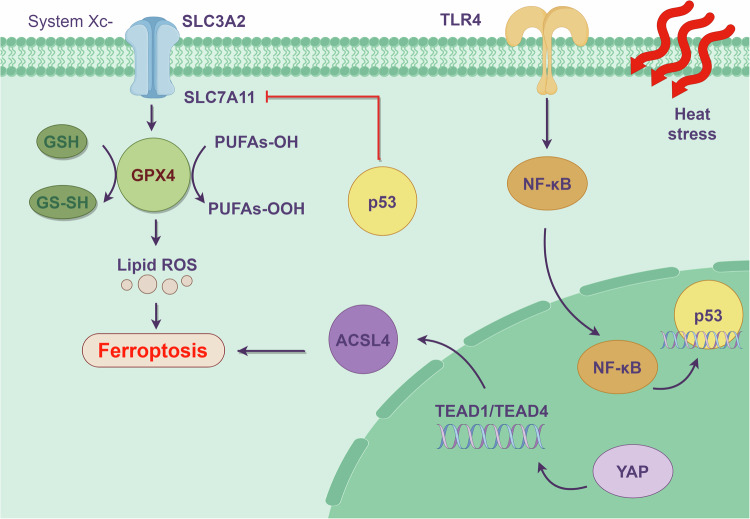
Signal transduction pathways in ferroptosis under HS. The occurrence of ferroptosis involves the transition metal iron, ROS, and PUFA-PLs. In a healthy body, System X_c_^-^ protects against oxidative stress damage, which consists of two subunits, SLC7A11 and SLC3A2. This system promotes the production of GSH by transporting cystine into cells and reducing it to cysteine. In the human body, GSH is a powerful reducing agent. As a cofactor of GPX4, it can convert reduced GSH into oxidized GSH and reduce lipid peroxides, thereby reducing oxidative stress damage. Therefore, System X_c_^-^ and GPX4 are important regulatory targets in ferroptotic amino acid metabolism. In a hyperthermic environment, p53 in the body increases significantly through the TLR4/NF-κB pathway. By acting on the SLC7A11 subunit of System X_c_^-^, the antioxidant system in the body is imbalanced, ultimately leading to the accumulation of lipid peroxidation and cell death. In addition, another pathway has been found to induce ferroptosis in EHS. The Hippo-YAP signaling pathway targets ACSL4 to promote ferroptosis. In previous studies, ACSL4 has been shown to contribute to the execution of ferroptosis by esterifying CoA to specific polyunsaturated fatty acids. Therefore, this pathway positively regulates ferroptosis and aggravates cell damage under HS.

Recent studies have demonstrated that, under HS, p53 can trigger ferroptosis by reducing the expression of SLC7A11 and affecting the functionality of System X_c_^-^ [117]. It seems that the expression of p53 is related to the level of ferroptosis [118]. Furthermore, Chen et al. [119]. discovered that the TLR4/NF-κB inflammatory signaling pathway appears to be connected to p53-mediated ferroptosis. Therefore, HS may induce ferroptosis through the TLR4/NF-κB/p53 signaling pathway (Fig. 3). Interestingly, although p53 appears to be an initiating target of ferroptosis, activation of p53 alone is not sufficient to directly induce ferroptosis. Only with the use of ferroptosis-inducing agents such as GPX4 inhibitors or high levels of ROS can the regulatory role of p53 be exerted [120].

Apart from p53, studies have shown that the role of acyl-CoA synthetase long-chain family member 4 (ACSL4) in ferroptosis has been confirmed [121] and was found to be a significant increase in radiational [122, 123] and ischemia/reperfusion-induced intestinal tissue [124]. Previous evidence suggests that ACSL4 is closely related to ferroptosis in rhabdomyolysis in exertional heatstroke. It seems to be Yes-associated protein-dependent via TEA domain transcription factor 1/TEA domain transcription factor 4 (TEAD1/TEAD4), thereby promoting lipid peroxidation [125] (Fig. 3). This suggests that ACSL4 may also be a key factor in ferroptosis in IEC.

#### Apoptosis

**Fig. 4:**
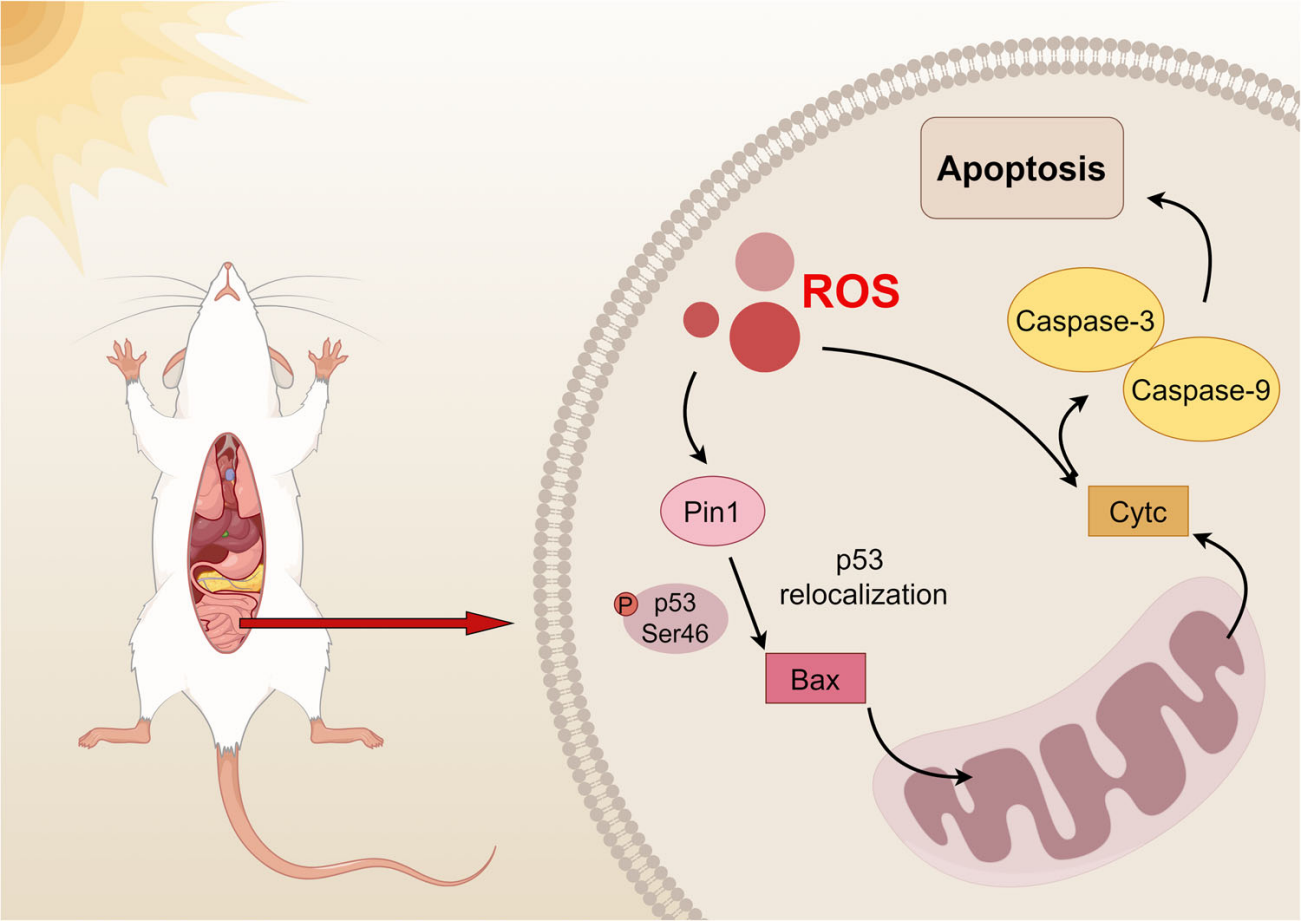
Mitochondria-mediated apoptosis under HS. Mitochondria-induced apoptosis plays an important role in cell death caused by heatstroke. In apoptosis, Bax serves as a start switch and is an executive protein in the mitochondrial regulation of cell death pathways. After the body is stimulated by high heat, Bax can be regulated by increased ROS. Studies have confirmed that ROS can promote the translocation of Bax and promote its conformational changes. This process is related to the phosphorylation of p53. Under HS, the massive production of ROS can facilitate p53’s phosphorylation at Ser46 and its entry into mitochondria via Pin1, which in turn encourages Bax to undergo a conformational shift and enter the mitochondria. Furthermore, ROS can also initiate the apoptotic pathway by directly causing Cytc to be released into the cytoplasm.

Three different pathways can cause the apoptotic process: the intrinsic pathway through mitochondria, the extrinsic pathway through death receptors, and the intrinsic pathway through the endoplasmic reticulum. In the HS model, the mitochondrial pathway has been extensively studied. One of the main reasons is that it is closely related to oxidative stress under HS. In a high-heat environment, the disruption of cellular oxidative balance leads to the massive generation of ROS. Research has demonstrated a correlation between ROS levels and apoptosis. One of the ways in which ROS promotes cell apoptosis is by exacerbating the conformational changes and translocation of the Bcl-2-associated X protein (Bax). Under HS, Bax shows significant growth and structural alterations. It has a strong connection to the elevated cytoplasmic Ca2^+^ concentration and the translocation of p53 to the mitochondria, both of which are brought on by the generation of ROS during heat stress [126]. Later research verified that ROS stimulates elevated phosphorylation of p53 at Ser46, which leads to interaction with prolylisomerase Pin1 with the movement of cytosolic p53 to mitochondria and upregulating Bax expression to induce apoptosis [127] (Fig. 4). The deletion of the p53 gene alleviates the damage of HS to endothelial cells and inhibits the mitochondrial apoptosis pathway, which in turn proves that p53 translocation plays a crucial role in HS-mediated apoptosis [128]. However, this study was limited to aortic endothelial cells, and whether its mechanism is also applicable to IEC remains to be confirmed by further experiments. In addition, excessive accumulation of ROS can induce mitochondria to release cytochrome c (Cytc) to promote apoptosis [129].

### Unbalanced flora in the gut

During HS, the intestinal flora is imbalanced, causing the overgrowth of harmful bacteria, especially gram-negative bacteria, while the number of *Lactobacillus* and *Bifidobacterium* is significantly reduced [130–132]. At the same time, the disorder of the metabolic function of the bacterial flora will also make the conditions more unfavorable [133]. In the gut, *Lactobacillus* and *Bifidobacterium* have various functions related to preserving intestinal stability. In normal conditions, they will spread across the mucus layer or stick to the surface of IECs, creating a bacterial membrane barrier that prevents harmful bacteria from adhering and colonizing [134, 135]. Furthermore, it has been demonstrated that *Lactobacillus* contributes to anti-inflammatory responses and promotes the growth of IECs [136]. They maintain the integrity of the intestinal barrier while guarding against the infiltration of inflammation. In the meantime, the p40 protein secreted by it protects IECs through the epidermal growth factor receptor [137]. Thus, it is obvious that decreasing probiotics damages the function of the intestinal barrier. In addition to the impact of microbiota remodeling, microbiota metabolites can also directly or indirectly affect the host’s physiological functions [138]. The metabolism of bile acids in the intestine and intestinal flora are tightly associated. Bile acids will build up in the intestines due to the decrease in beneficial bacteria, finally causing damage [39, 139]. In summary, intestinal function is directly influenced by the remodeling of the microbiota after HS. Furthermore, the damage to the intestine is made worse by the colonization of harmful bacteria.

In addition to bacterial communities, fungal communities are also an important component of the intestinal microbiome. Although they only account for 1% of the total microbiome, they have an impact on various physiological and pathological processes [140]. There are two types of fungi in the body: symbiotic fungi and pathogenic fungi. When the body is subjected to stimulation such as HS, due to the host’s decreased immunity or changes in the microenvironment, the over-proliferation of certain conditional pathogenic fungi can lead to a serious imbalance of the host’s intestinal microorganisms, which can not only cause intestinal diseases but also induce diseases outside the intestine [141, 142]. Thus, fungal dysbiosis, characterized by a decrease in fungal diversity and an increase in certain opportunistic pathogens, such as Candida albicans and Malassezia [143], has been reported in diseases such as IBD [144], colorectal cancer [145], and sepsis [146]. Besides, after HS, mitochondrial disturbances caused by oxidative stress may put patients in a state of hypoglycemia [147, 148]. Abnormal blood sugar levels can lead to increased ketone body synthesis in the liver, making patients more susceptible to Candida albicans infection and manifestations of Candida sepsis [146]. Although the contribution of Candida albicans to the pathogenesis of such diseases is not fully understood, it is currently believed that it mainly plays a role by driving Th17 cell-mediated immune responses and disrupting the homeostasis of the intestinal microbiome [149]. Furthermore, candida albicans can directly damage the epithelial membrane, triggering danger response signaling pathways and causing epithelial immunity [150]. Another risk factor for pathogenic fungal colonization is the use of antimicrobial agents; studies in mice have shown that long-term oral antifungal medications can exacerbate colitis and worsen allergic airway disease [151]. The mechanism may be related to the mutually beneficial ecological niche between bacteria and fungi. The disruption of fungal communities can also stimulate the growth of bacterial pathogens, while the depletion of short-chain fatty acid-producing bacteria will weaken their inhibitory effect on Candida albicans, ultimately leading to the development or worsening of intestinal inflammation [152]. Consequently, we need to be cautious about anti-infection treatment after HS. In future studies on the mechanism of HS, the crosstalk between fungi and bacteria should also be considered.

## Potential therapeutic strategies for intestinal injury after HS

### Maintenance of the intestinal barrier

After HS, intestinal endotoxins are translocated into the blood, making anti-inflammatory treatment more difficult [78]. Hence, it is very important to maintain the integrity of the intestine. A study found that dexmedetomidine can maintain the integrity of the intestinal barrier by maintaining the expression of TJ proteins and reducing intestinal apoptosis. At the same time, it can also reduce inflammation and prevent multiple organ dysfunction in mice with HS by inhibiting the activation of the intestinal NF-κB pathway. Therefore, in addition to its well-known analgesic and sedative properties, dexmedetomidine has other protective effects [153]. What’s more, intestinal alkaline phosphatase [154], amino acids [155, 156], misoprostol [67], polyunsaturated fatty acids [157], etc., have also been found to play a protective role by increasing the expression of TJ proteins and can be used as a potential means of intestinal protection during HS. It should also be noted that excessive ROS under HS can also damage the intestinal epithelium. According to Li Li et al., a significant reversal of necroptosis in intestinal epithelial cells was observed in animals and cells pretreated with the ROS scavenger *N*-acetylcysteine [66]. In short, inhibiting excessive ROS is the key to the treatment of HS. Antioxidants such as _L_-carnitine or vitamin C can improve intestinal damage after HS [158–160]. This may be related to anti-oxidation and anti-cell death. Additionally, they also have a good anti-inflammatory effect. This indirectly shows that oxidative stress is one of the important mechanisms of HS damage. Although antioxidants can improve the damage of HS, the underlying mechanism of intestinal homeostasis is still unclear [161–163] (Table 1).

**Table 1:** Potential therapeutic strategies and their effects on the gut.

Name	Intestinal integrity	Immunomodulation	Other effect(s)	Ref.
Dexmedetomidine	Intestinal permeability↓ Occludin↑ ZO-1↑ Morphological damage↓	TNF-α ↓ IL-6↓ IL-1β ↓ NF-κB activation↓	Bax↓ Bcl-2↑	[153]
Intestinal alkaline phosphatase		IL-1β ↓ IL-6↓ TNF-α ↓	—	[154]
Five amino acids oral rehydration solution	Na^+^ absorption↑ Villus height↑ Intestinal permeability↓ Occludin↑ Claudin-1↑ Claudin-5↑ Claudin-2↓	—	—	[155]
_L_-Arginine	ZO-1↑ Claudin-1↑ Morphological damage↓ Intestinal permeability↓ Villus/crypt ratio↑	AMPK activation↑ Serum cortisol level↓	HSF1↓ HSP expression↑ Caspase-3↓	[156]
Misoprostol	Intestinal permeability↓ Claudin-1↑	COX-2↓ ERK1/2↓	Oxidative stress↓ Intestinal apoptosis↓ HSP90↓ Mucin-2↑	[67]
Eicosapentaenoic acid	Intestinal permeability↓ Morphological damage↓ Occludin↑ ZO-1↑	—	—	[157]
*N*-acetyl-l-cysteine	Morphological damage↓	—	ROS↓ MDA↓ SOD↑ RIPK1-RIPK3 complex formation↓ Mitochondrial depolarization↓	[66]
_L_-carnitine	Morphological damage↓	IL-1β↓ IL-2↓ IL-6↓ TNF-α↓ IL-10↑ NF-κB activation↓	Treg cells↑ Macrophages↑ Th17 cells↓	[158]
Vitamin C	Goblet cells↑ Villus height↑ Morphological damage↓	TNF-α↓ IL-10↑ NF-κB activation↓	SOD↑ CAT↑ GPx↑	[159, 160]
α-Lipoic acid	TEER↑	TGF-β↓ COX-2↓	HSP70↑ Nrf2 expression and nuclear translocation↓	[161]
Resveratrol	ZO-1↑ Occludin↑ Intestinal permeability↓	—	MDA↓ GSH↑ SOD↑ CAT↑ ROS↓ HO-1 activation↑ PKC activation↓	[162, 163]
Probiotic	Occludin↑ Zonulin↓ ZO-1↑ Morphological damage↓ Goblet cells↑ Intestinal permeability↓	IL-6↓ IFN-γ↓ TNF-α↓ NF-κB activation↓	Stabilize the intestinal microflora Intraepithelial lymphocyte count↓ Regulatory T cell count↑	[130, 164–169]
Mesenchymal stem cell	Morphological damage↓	IL-1β↓ IL-6↓ TNF-α↓ IL-10↑	—	[171]

Upwards arrow: Increase or enhancement; downwards arrow: Decrease or inhibition.

*TNF* tumor necrosis factor, *IL* interleukin, *NF-κB* nuclear factor κ-light-chain-enhancer of activated B cells, *Bax* B-cell lymphoma-2 assaciated X protein, *Bcl-2* B-cell lymphoma-2, *AMPK* adenosine 5-monophosphate-activated protein kinase, *HSF* heat shock factor, *HSP* heat shock protein, *caspase* cysteinyl aspartate specific proteinase, *COX* cyclooxygenase, *ERK* extracellular regulated protein kinases, *ROS* reactive oxygen species, *MDA* malondialdehyde, *SOD* superoxide dismutase, *RIPK* receptor-interacting serine/threonine-protein kinase, *CAT* catalase, *GPx* glutathione peroxidase, *TEER* trans-epithelial electrical resistance, *TGF* transforming growth factor, *Nrf* nuclear respiratory factor, *GSH* glutathione, *HO-1* heme oxygenase 1, *PKC* protein kinase C, *IFN* interferon.

### Regulation of microbial community homeostasis

Alterations in the composition of the intestinal microbiota increase the likelihood of opportunistic enteric infections [164]. Therefore, effective maintenance of the homeostasis of the microbiota has gained recent interest. A clinical study shows that dietary supplementation with probiotics can effectively alleviate intestinal damage following exercise in hot conditions [165]. Likewise, prebiotics also have a strong impact on preventing HS-induced intestinal damage [166]. They can protect the intestine through a variety of mechanisms, such as upregulating TJ expression [130], reducing the concentration of zonulin [167], and lessening the intestinal morphological alterations exposed to HS [168]. As a result, it can successfully reduce bacterial turbulence and lipopolysaccharide penetration. Additionally, they can also achieve anti-inflammatory function by inhibiting NF-κB pathways and participating in immune regulation [169] (Table 1). Hence, stabilization of the gut microbiota composition is considered an effective strategy to improve gut health and protect the intestines against heat stress [166].

### The therapeutic potential of mesenchymal stem cells

Nowadays, MSCs are widely used in the treatment of various diseases because they can participate in the body’s immune regulation through cell contact-dependent mechanisms or secrete paracrine factors [170]. Treatment with MSC can improve intestinal damage after HS, specifically by improving intestinal mucosal edema, necrosis, and villus shedding, and reducing the inflammatory response of intestinal tissue. The therapeutic effect is due to reducing the level of chemokines and thus inhibiting local inflammation (Table 1). In addition, it may regulate the body’s immune function by affecting the differentiation and expression of T cells [171, 172]. In conclusion, MSC provides new theories and methods for the treatment of HS, but the specific treatment plans and mechanism research still needs to be further improved.

## Conclusions

This review summarizes the progress of the intestinal damage mechanisms caused by HS. HS is considered an important and common stressor that has increasingly attracted public health attention. However, due to the complexity of its pathogenesis, there are no effective treatment measures in clinical settings, which has caused HS to become a high-mortality disease. Notably, as the main organ of HS damage, the intestinal tract assumes a launching role that cannot be ignored in SIRS. Under HS conditions, the integrity of the intestinal tract is destroyed, followed by the general inflammation response of the body, which is a key event for the body’s disease. Intestinal injury caused by HS depends on the participation of various signal pathways and disease models. At the same time, its pathological damage is not limited to damage to epithelial cells. Although the mechanisms of intestinal damage caused by HS are diverse and complicated, the common effect is damage to the intestinal barrier. Therefore, it is necessary to pay attention to the protection and repair of intestinal barriers during the HS treatment process. Based on the current mechanism research, the corresponding therapeutic drugs have achieved considerable progress, for example, probiotics, antioxidants, and amino acids. Even so, the prevention of HS is more effective than treatment. Future research needs to focus on the cellular and molecular pathways that act behind HS. At present, only a few mechanisms, such as HSR and the oxidation stimulus reaction, have been clearly described. There are still some gaps in the remaining knowledge. In order to have a better basis and direction for clinical treatment, we still need more research.

## Data Availability

Data sharing is not applicable to this article as no new data were created or analyzed in this study.
